# Metabolites from Marine Microorganisms, Micro, and Macroalgae: Immense Scope for Pharmacology

**DOI:** 10.3390/md17080464

**Published:** 2019-08-08

**Authors:** Noora Barzkar, Saeid Tamadoni Jahromi, Hadi Bolooki Poorsaheli, Fabio Vianello

**Affiliations:** 1 Department of Marine Biology, Faculty of Marine Science and Technology, University of Hormozgan, Bandar Abbas 74576, Iran; 2 Persian Gulf and Oman Sea Ecology Research Center, Iranian Fisheries Sciences Research Institute, Agricultural Research Education and Extension Organization (AREEO), Bandar Abbas 93165, Iran; 3 Road, Housing & Urban Development Research Center (BHRC), Persian Gulf Branch, Bandar Abbas 93144, Iran; 4 Department of Engineering, Islamic Azad University, Bandar Abbas 1696, Iran; 5 Department of Comparative Biomedicine and Food Science, University of Padua, viale dell’Università 16, Legnaro 35020, Italy

**Keywords:** marine microorganisms, natural products, pharmaceutical potential, bacteria, microalgae, macroalgae

## Abstract

Marine organisms produce a large array of natural products with relevance in drug discovery. These compounds have biological activities such as antioxidant, antibacterial, antitumor, antivirus, anticoagulant, anti-inflammatory, antihypertensive, antidiabetic, and so forth. Consequently, several of the metabolites have made it to the advanced stages of clinical trials, and a few of them are commercially available. In this review, novel information on natural products isolated from marine microorganisms, microalgae, and macroalgae are presented. Given due research impetus, these marine metabolites might emerge as a new wave of promising drugs.

## 1. Introduction

Oceans cover about 70% of the earth’s surface, serving as the habitat of a great diversity of organisms [1]. These organisms produce numerous metabolic products. Especially, lower organisms elaborate a multitude of secondary metabolites as signaling molecules for “defense and offense”. These compounds, which belong to diverse chemical classes, can act as potential therapeutics for healthcare [2]. In the past decades, several promising therapeutics have been extracted from bacteria, fungi, corals, micro- and macroalgae, gorgonians, sponges, nudibranchs, bryozoans, sea cucumbers, tunicates, and sea hares, among other marine organisms [3]. Considerable efforts have been directed towards the isolation of these compounds, and at the moment, more than 10,000 natural products (NPs) of potential biotechnological interest have been isolated [4]. The present review reports on the most promising bioactive compounds of marine origin, emphasizing their pharmaceutical potential.

## 2. Bioactive NPs from Marine Bacteria and Fungi

In the marine environment, bacteria and fungi are pervasive. In the past decades, the number of reported bioactive compounds derived from marine bacteria and fungi has steadily increased [5]. Marine bacteria produce a large repertoire of secondary metabolites to survive in the hostile oceanic conditions. Among others, thermophilic and archaea bacteria elaborate thermostable enzymes which belong to diverse classes [1,6].

### 2.1. Antibiotic Activity

**Figure 1:**
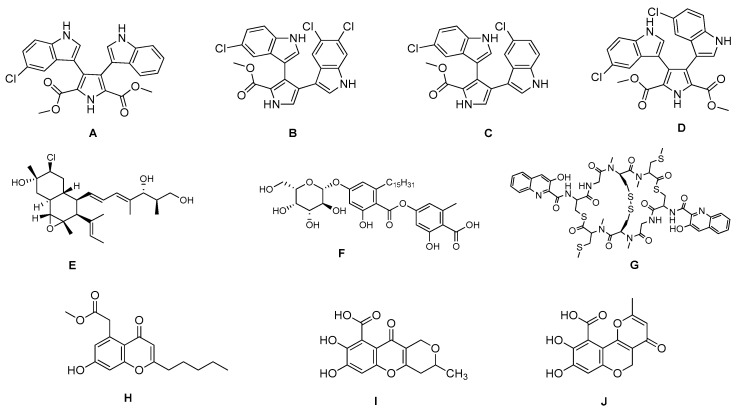
Chemical structure of marine natural compounds isolated from marine microorganisms. (A) Lynamicin E; (B) Lynamicin B; (C) Lynamicin A; (D) Lynamicin D; (E) Dehydroxychlorofusarielin B; (F) Aquastatin A; (G) Thiocoraline; (H) Pestalotiopsone F; (I) Anhydrofulvic acid; (J) Citromycetin.

The myxomycetes *Lycogala epidendrum* produces halogenated bisindole pyrrole derivatives (Lynamicins A–E) (Figure 1A–D) with antibacterial activity against *Enterococcus faecalis*, *Staphylococcus epidermidis*, and *Staphylococcus aureus*. Efficacy against these pathogens suggests the potential application of these compounds for the treatment of nosocomial infections [7]. Similarly, a bacterium isolated from the sea grass *Thalassia* produces a highly brominated pyrrole antibiotic [8]. The crude extract of *Nocardia* sp. strain, isolated from the marine red marine alga *Laurenica spectabilis,* produces active compounds against bacterial and fungal pathogens [9]. The eggs of the oriental shrimp *Palaemon macrodactylus* harbor bacterial epibionts with antifungal potential towards the pathogenic fungus *Lagenidium callinectes* [10]. Likewise, marine fungi have been studied for their bioactive compounds and they have proven to be a valuable source of antibacterial, antibiotic, antifungal, and anticancer compounds [11]. A marine *Aspergillus* sp. fungus was isolated from the large marine brown alga *Sargassum horneri,* and it produces a polyoxygenated decalin, dehydroxychlorofusarielin B (Figure 1E), which has demonstrated antibacterial activity against methicillin-resistant and multidrug-resistant (MDR) *S. aureus* [12]. Another fungal *Aspergillus* sp. strain, isolated from a sea fan (*Alcyonacea*), was found to produce antibacterial compounds against *S. aureus* ATCC 25923 and methicillin-resistant *S. aureus* [13].

### 2.2. Anticancer Activity

Besides antibiotics, marine microorganisms are also a source of anticancer principles. The bacterium *Micromonospora* sp. produces thiocoraline (Figure 1G), a depsipeptide which inhibits cellular DNA polymerase-α. This substance has been applied for the treatment of cancer in preclinical research [14]. An unidentified fungus of the *Pleosporales* order (strain CRIF2) produces several compounds showing weak cytotoxic activity against tumor cell lines [15]. A *Pestalotiopsis* sp. fungus, isolated from the leaves of *Rhizophora mucronata,* produces a chromone, namely, pestalotiopsone F (Figure 1H), which displays cytotoxic activity against L5178Y murine cancer cells [16].

### 2.3. Antidiabetic Activity

Diabetes mellitus (or diabetes) is a debilitating and often life-threatening disorder that is prevalent worldwide, and the number of patients is significantly increasing [17]. Marine fungi have been screened for possible antidiabetic compounds [18]. The *Cosmospora* sp. SF-5060 fungus produces aquastatin A (Figure 1F), a secondary metabolite which inhibits protein tyrosine phosphatases 1B (PTP1B) with an effective concentration (EC_50_) value of 0.19 µM. As PTP1B regulates insulin signaling and leptin receptor, aquastatin A might find an application in diabetes management [18]. Another marine fungus, *Penicillium* sp. JF-55, produces methylethylketone, which exerts inhibitory action on PTP1B as well. Other substances obtained from marine fungi are penstyrylpyrone, anhydrofulvic acid (Figure 1I), and citromycetin (Figure 1J), all displaying inhibitory actions of PTP1B with IC_50_ values in the micromolar range [19].

Generally, marine bacteria and fungi species survive under hostile conditions, for example, high shear stress, high salinity, high light intensity, and low temperatures, which result in the elaboration of a large array of fascinating and structurally complex molecules. In Table 1, some of the bioactive compounds produced by marine fungi and bacteria are listed.

## 3. Metabolites with Potential Beneficial Activities from Marine Algae

Marine algae are the primary producers of oxygen in the aquatic environment and sit at the bottom of the marine food chain, serving all other organisms [31]. Marine algae can be divided into two main groups: macroalgae (seaweeds) and microalgae, both being prolific sources of bioactive substances [32]. Therefore, research is in progress for evaluating their medicinal prospects.

### 3.1. Marine Microalgae: Blue-Green Algae (Cyanobacteria)

Marine microalgae typically constitute the phytoplanktons. They can be categorized into three groups: blue-green algae (Cyanobacteria), diatoms (Bacillariophyta), and dinoflagellates (Dinophyceae). There are over 50,000 different species of microalgae, of which only a few have been characterized [18]. Significant biochemical differences have been found among marine microalgae, resulting in a broad spectrum of novel bioactive compounds [33] of pharmaceutical interest [34]. Some of them show high antiviral and anti-HIV activity [35,36]. Recently, a new natural anti-AIDS drug has been derived from *Lyngbya lagerhaimanii* and *Phormidium tenue* (Table 2). Calcium spirulan isolated from *Spirulina platensis* possesses strong antiviral activity [37]. Some cyanobacteria strains produce antifouling compounds with antibiotic activity [38]. For example, the extracts of *Lyngbya majuscule* have been tested as a potential source of antifouling agents [39]. Some of the cyanobacteria-derived products have multiple properties. For example, ulithiacyclamide and patellamides A and C are known for their antimalarial, antitumor, and MDR-reversing activities [40]. Two new bioactive compounds, dolastatin 13 (Figure 3C) and lyngbyastatins 5–7 (Figure 3D), were isolated from *Lyngbya* spp., which inhibited elastase from porcine pancreas, with an IC_50_ = 3–10 nM [41]. Of the three abovementioned groups, cyanobacteria have been credited with the most bioactive compounds. Cyanobacteria (*Cyanophyta*) are a group of Gram-negative bacteria and one of the richest sources of novel bioactive compounds with antifungal, anti-inflammatory, antibiotic, and antitumor activities (Table 1), which make them interesting candidates for the production of molecules for new potential pharmaceutical applications [42].

#### 3.1.1. Antibiotic Activity

**Figure 2:**
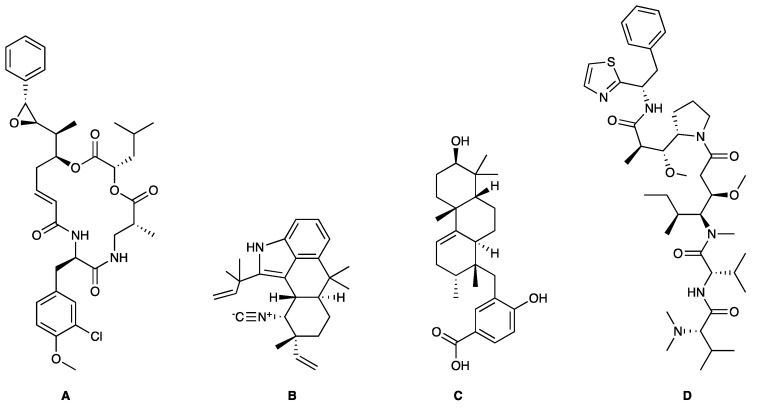
Chemical Structure of marine natural compounds isolated from marine microalgae. (A) Cryptophycin; (B) Ambiguine H isonitriles; (C) Noscomin; (D) Dolastatin 10.

The antibacterial activity of cyanobacteria might make them useful antibiotics sources. For example, the extracts of *Anabaena variabilis* and *Synechococcus elongates* inhibit the growth of *Escherichia coli*, *Enterococcus*, and *Klebsiella* [43]. The extracts of *Synechocystis* sp. and *Synechococcus* sp. showed significant antimicrobial activity towards Gram-positive bacteria [44]. Noscomin (Figure 2C), a diterpenoid, was isolated from *Nostoc commune* and possesses good antibacterial properties [45]. Other antibiotics are malyngamides (malyngamide D and malyngamide D acetate) and amides of the fatty acid (8)-7(S)-methoxytetradec-4(E)-enoate, isolated from the *L. majuscule* [46]. Analogously, ambiguine H isonitrile (Figure 2B) and ambiguine I isonitrile are antibiotic alkaloids purified from *Fischerella* sp. [47]. Another example of an antibacterial compound is kawaguchipeptin B, isolated from the toxin-producing cyanobacterium *Microcystis aeruginosa* [48].

#### 3.1.2. Antitumor Activity

Antitumor compounds affecting cell signaling by the activation of the protein kinase C cascade have been demonstrated in cyanobacteria [49]. Cyanobacteria elaborate anticancer compounds, such as dolastatin 10 (Figure 2D), curacin A, and cryptophycin (Figure 2A), which target tubulin or actin filaments of eukaryotic cells. Dolastatin 10, a strong microtubule inhibitor that can arrest cell mitotic division, was isolated from *Symploca* sp. [50]. Curacin A was isolated from *L. majuscule* and is a strong antiproliferative agent, inhibiting microtubule assembly [51]. Cryptophycin was isolated from marine *Nostoc* sp. GSV 224 and is an anticancer drug candidate with efficacy against L1210 leukemia cells, ovarian carcinoma cells, and drug-resistant breast cancer cells [52]. The mechanism of action of cryptophycin involves binding at the microtubule ends, leading to the disruption of cell mitosis [53]. Odoamide is a newly discovered cyclic depsipeptide from *Okeanis* sp., showing strong cytotoxicity against HeLa S3 human cervical cancer cells (IC_50_ = 26.3 nM) [54]. Hierridin B from *Cyanobium* sp. LEGE 06113 exerted cytotoxicity towards HT-29 colon adenocarcinoma cells [55].

#### 3.1.3. Antifungal Activity

**Figure 3:**
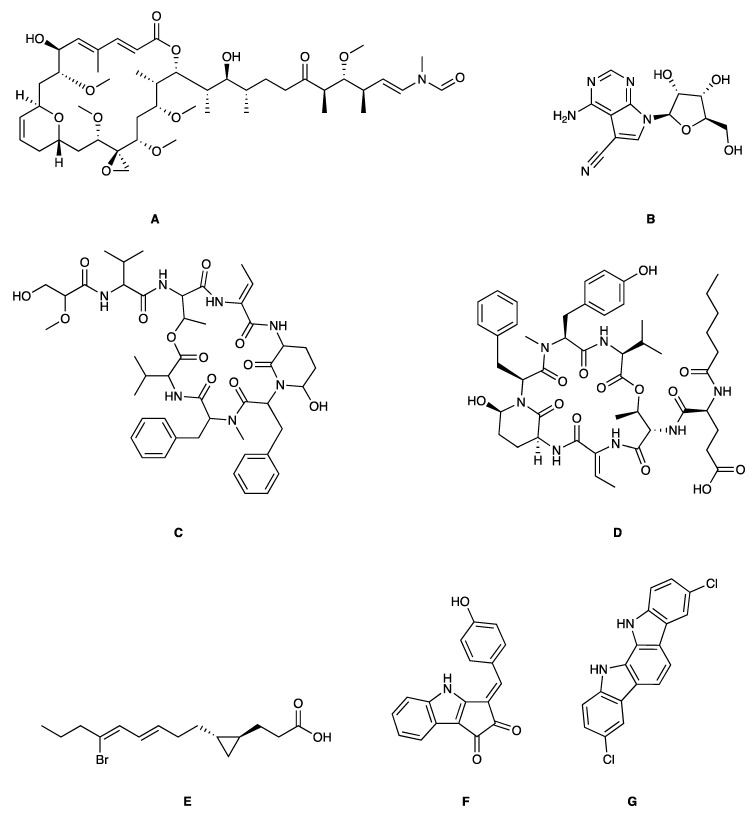
Chemical Structure of marine natural compounds isolated from marine microalgae. (A) Tolytoxin; (B) Toyocamycin; (C) Dolastatin 13; (D) Lyngbyastatin 7; (E) Majusculoic acid; (F) Nostodione A.; (G) Tjipanazole D.

Cyanobacteria are important producers of antifungal substances as well, as they are competitors and predators of parasitic fungi. Many antifungal compounds have been isolated from marine extracts of cyanobacteria, including hapalindoles, tolytoxin (Figure 3A), 7-OMe-scytophycin-B, toyocamycin (Figure 3B), tjipanazole D (Figure 3G), hassallidin A, nostocyclamide, and nostodione A (Figure 3F) [56]. 7-OMe-scytophycin-B, a metabolite isolated from marine *Anabaena* sp. HAN21/1, has shown activity against *Aspergillus flavus* and *Candida albicans* [57]. A new antifungal lactone compound, majusculoic acid (Figure 3E), has been isolated from a marine cyanobacterial mat community. Majusculoic acid displayed antifungal activity towards *C. albicans* ATCC 14503 [58]. The butanol extracts of *Spirulina* sp. exhibited antifungal activity against *Candida glabrata* [59].

#### 3.1.4. Antimalarial Activity

Antimalarial activity of natural products isolated from cyanobacteria has been reported. Gademann and Kobylinska (2009) isolated an acyl proline derivative, tumonoic acid I, from *Blennothrix cantharidosmum*, which exhibited moderate toxic activity against *Plasmodium falciparum* (IC_50_ = 2 μM) [60]. Two new antimalarial depsipeptides, companeramides A and B, have been extracted from a marine *Panamanian* cyanobacteria assemblage [61]. *Oscillatoria nigro-viridis* produces two new linear peptides, viridamides A and B, with antitrypanosomal and antileishmanial activity [62]. Moreover, cyanobacteria are sources of vitamins B and E [63]. Pigments extracted from cyanobacteria, such as carotenoids and phycobiliproteins, are already industrially applied as food coloring additives, as supplements for health and fertility of dairy cattle, and in the cosmetics industry.

#### 3.1.5. Anti-inflammatory Activity

Cyanobacteria metabolites have also shown pronounced anti-inflammatory effects. For instance, *bis*-bromoindoles from *Rivularia* sp. displayed powerful anti-inflammatory activity [8]. An anti-inflammatory compound malyngamide F acetate has been derived from *L. majuscule*. This substance inhibited the production of nitric oxide (NO) in stimulated RAW 264.9 cells [64] by blocking the MyD88 inflammation pathway.

### 3.2. Marine Macroalgae

Macroalgae, or seaweeds, are found in intertidal regions and tropical waters. They are multicellular organisms with various arrays of morphological types and sizes and can be further classified by their photosynthetic pigments into red algae (Rhodophyceae), green algae (Chlorophyceae), and brown algae (Phaeophyceae) [65]. Currently, over 3200 novel products have been extracted from macroalgae, the majority of which come from subtropical and tropical waters [66]. Compounds with medical applications, such as antitumor, antioxidant, antiviral, antifouling, anticoagulant, antibacterial, antifungal, and anthelminthic activities, have been detected in macroalgae [67,68]. Red seaweeds are proposed as anticoagulants, anthelmintic, and in the treatments of gastritis and diarrhea [69]. The traditional medical uses of green seaweed spans form anthelmintic to astringent and anti-gout. Brown seaweeds are applied to cure rheumatic diseases, hypertension, arteriosclerosis, menstrual disorders, skin diseases, gastric ulcers, goiter, and syphilis and are also used as anticoagulants. Polysaccharides, such as ulvans from green seaweeds, alginates, fucans, laminarin from brown seaweeds, and carrageenans and porphyrans from red seaweeds, can stimulate defense responses against plant pathogens [70]. Thus, marine algae yield a large diversity of bioactive metabolites and appear to be a potential resource of interesting pharmacological substances. The sections below present the therapeutic compounds and functions of members from each of the three groups of seaweeds.

#### 3.2.1. Red Seaweeds

**Figure 4:**
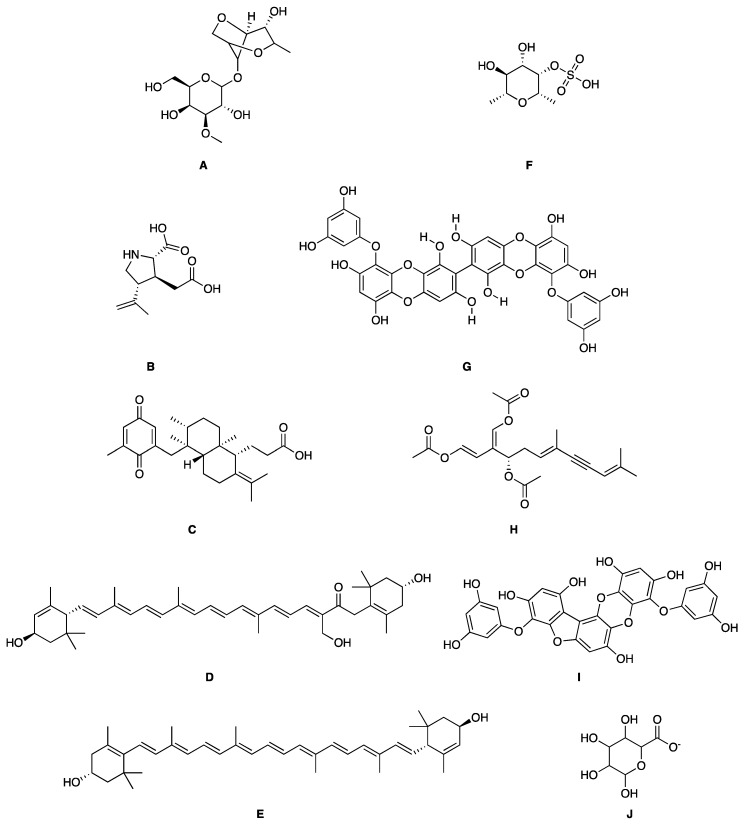
Chemical structure of marine natural compounds isolated from marine macroalgae. (A) Agar; (B) Alpha-Allokainic acid; (C) Stypoquinonic acid; (D) Siphonaxanthin; (E) Lutein; (F) Fucoidan; (G) 8,8’-Bieckol; (H) Caulerpenyne; (**I**) Phlorofucofuroeckol A; (**J**) Sodium alginate.

Red seaweeds are commonly considered beneficial for human health and an important source of bioactive compounds [86]. For centuries, their extracts have been applied for the cure of asthma, thyroid goiter, urinary infections, stomach ulcers, and even tumors. Among the compounds isolated from red seaweeds, sulfated polysaccharides are economically the most important bioactive compound because of their wide application in medicine. These polysaccharides are carrageenan, agar (Figure 4A), agarose, and furcellaran (Danish agar). Carrageenan is produced by the genera *Chondrus, Eucheuma, Gigartina*, and *Iridea* and is considered an effective remedy for gastric and duodenal ulcers [87]. *Chondrus crispus* is a good source of carrageenan that has an antiviral property, in particular against influenza B and mumps virus [88]. Agar and agarose are used for interferon production, usage as antiviral compounds, and improving B- and T-cell activity [89]. A polysaccharide (Mw = 100–500 kDa) isolated from the fermented red seaweed *Lomentaria catenata* possesses anticoagulant activity [90]. Fucoidan (Figure 4F), extracted from *Gracilaria corticata,* showed activity against both colorectal and breast cancer [91]. An anthelmintic compound, L-α-kainic acid (Figure 4B), has been isolated from *Digenea simplex* [92]. Deepa et al. (2017) reported on the possible effects of *G. corticata* on cancer treatments, inflammation, and infectious diseases [91]. Some red seaweeds, such as *Rhodomela confervoides, Symphyocladia latiuscula*, and *Polysiphonia urceolata,* produce phenolic compounds, which have shown antidiabetic activity. These compounds possess the capacity to inhibit protein tyrosine phosphatase (PTPase), which is responsible for the response to insulin. Collins reported on the antiasthmatic activity of polyphenolic extracts of *Laurencia undulate* [68]. A new potent inhibitor of lipoxygenase (LOX), which plays a crucial role in neurodegeneration, has been isolated from *Odonthalia corymbifera* [93]. The substance is pheophytin A, which can be applied as a new therapeutic, and is considered an excellent opportunity for the treatment of neuropathologies such as Alzheimer’s disease.

##### Antiviral Activity

Witvrouw et al. (1994) isolated a polysaccharide, galactan sulfate, from *Agardhiella tenera* that showed activity against HIV-1 and HIV-2, with IC_50_ values of 0.5 and 0.05 μg/L, respectively [94]. Devi et al. reported on the antioxidant and antimicrobial activity of the methanolic extracts of different Indian red seaweeds [95]. An antiviral compound, sulfated xylomannan, has been extracted from the Indian red seaweed *Scinaia hatei* that inhibited HSV-1 and HSV-2 (IC_50_ = 0.5–1.4 μg/mL) [96]. Water-soluble polysaccharidic extracts of *Sphaerococcus coronopifolius* and *Boergeseniella thuyoides* collected from the coast of Morocco showed antiviral properties against viruses, including HIV and HSV-1 [97]. Serkedjieva (2004) extracted a bioactive compound from *Ceramium rubrum* isolated from the Black Sea. This metabolite inhibited types A and B influenza viruses, both in vivo and in vitro, followed by the reduction of cytopathogenic effects [98].

##### Antioxidant Activity

Antioxidant properties has been found in several species of red seaweeds, including *Gracilaria, Halymenia, Laurencia, Ahnfeltiopsis*, and *Polysiphonia*. For example, mycosporine-like amino acids isolated from *Ahnfeltiopsis devoniensis* show antioxidant activity [99]. Moreover, two classes of natural compounds, polyphenols and bromophenols, with known antioxidant activity were isolated from red seaweeds [100]. The ethanolic [101] and methanolic [102] extracts isolated from *Gracilaria tenuistipitata* showed antiproliferative activity on the oral carcinoma cell line *Ca 9-22* by modulating oxidative-stress-induced cell apoptosis.

##### Antibiotic Activity

Rahelivao reported on the properties of crude extracts of the red algae *Laurencia complanata,* which displayed antibacterial activity against *Streptococcus pneumoniae, Bacillus cereus*, and *S. aureus* [81]. Four tetracyclic brominated 1, 4-diterpenes were isolated from the extract of *S. coronopifolius*, collected from the rocky coasts of Corfu Island (Greece). These diterpenes showed antibiotic activity against a panel of bacteria, including methicillin-resistant *S. aureus* (MRSA) and MDR strains, with MIC (Minimum Inhibitory Concentrations) values in the 16–128 µg/mL range [103]. Crude methanolic extracts isolated from *Acanthaphora spicifera* showed antibacterial activity against *E. coli*, *Bacillus subtilis*, *Pseudomonas aeruginosa*, and *Bacillus palmitus* [104]. Also, antifungal activity of the extracts against *C. albicans*, *Aspergillus niger*, and *Microsporum gypseum* was observed [105].

##### Antitumor and Anticoagulant Activities

A novel polyhalogenated monoterpene, halomon, isolated from *Portieria hornemannii*, shows cytotoxic action against numerous human tumor cell lines (brain, kidney, and colon) and is currently in the preclinical testing phase [106]. Moreover, Andrianasolo (2006) isolated three halomon derivatives from *P. hornemannii*, which exhibited inhibitory effects on the DNA methyltransferase-1 enzyme [107]. Among other functions, DNA methyltransferase has a profound role in epigenetics and gene expression. The anticoagulant activity of sulfated polysaccharides derived from *Delesseria sanguinea* (*Ceramiales*) [108] and a sulfated galactan fraction from *Botryocladia occidentalis* (*Rhodymeniales*) was reported [109]. Matsuhiro et al. (2005) reported on an antiviral sulfated galactan from *Schizymenia binderi* [110]. Moreover, *Botryocladia leptopoda* extracts exhibited stimulant activities on the central nervous system (CNS) in a mouse model [111].

#### 3.2.2. Brown Seaweeds

Brown seaweeds contain several pigments, such as fucoxanthin, violaxanthin, and β-carotene [112]. Fucoxanthin occurs in edible marine brown algae, including *Undaria pinnatifida*, *Laminaria japonica, Sargassum fulvellum*, and *Hijikia fusiformis*, and possesses antioxidant, anticancer, antiobesity, antidiabetic, and antiphotoaging activities [113]. The ethanolic extract of *Turbinaria conoides* demonstrated antioxidant, antibacterial, and anticancer properties. The sources and properties of some bioactive principles in the extracts of brown seaweeds are reported in Table 2. For example, organic solvent extracts from *Sargassum wightii* and *Sargassum ilicifolium* exhibited an interesting anticancer activity on several cells lines [114]. Several studies on *U. pinnatifida* demonstrated anti-hyperglycemic, antitumor, anti-hypertensive, and antiobesity potential [115]. Khan reported on an omega-3 essential fatty acid, stearidonic acid, isolated from *U. pinnatifida* which showed activity against erythema, mouse ear inflammation, edema, and blood flows (IC_50_ = 160, 314, and 235 μg/per ear, respectively) [116]. Laminarin is a water-soluble polysaccharide found in brown algae that has strong heparin-like activity and, therefore, is useful as an anticoagulant, antilipemic, antiviral, or anti-inflammatory agent [17]. Park et al. (2011) reported that that fucoidan reduces lipid accumulation by stimulating lipolysis, and it can be beneficial for obesity therapy [117]. Spavieri reported on the antimycobacterial, antiprotozoal, and cytotoxic activity of 21 brown algae (*Phaeophyceae*) from British and Irish waters [118]. Several other bioactive compounds were reported for fucoidans isolated from different species of brown algae, including antiviral and antibacterial activities [119]. Moreover, fucoidans isolated from brown seaweed species present immunomodulating activity, involving the increased generation of macrophage-mediated responses such as interleukin-2 (IL-2), interleukin-12 (IL-12), and interferon gamma (IFN-γ) [120]. Salgado reported on the interactions between polyphenolic compounds from the brown alga *Padina gymnospora* and cell wall alginates, leading to the absorption of ultraviolet radiation [121]. Also, diekol isolated from *Ecklonia cava* showed antifungal, anti-inflammatory, and anti-type-II diabetes activities in mouse models [122].

##### Antibacterial and Antioxidant Activities

The essential oil derived from *U. pinnatifida* possesses potent antibacterial and antioxidant activities [114]. The methanolic extracts of *Sargassum platycarpum* A and *Sargassum latifolium* B were shown to be highly effective against Gram-positive bacteria [123]. Three novel products from *Ecklonia maxima*—eckol, dibenzo-dioxine-2,4,7,9-tetraol, and phloroglucinol—were shown to exhibit free-radical scavenging activity by a DPPH assay, and the EC_50_ values were 0.008, 0.012, and 0.128 μM, respectively [124]. The phenolic extracts of *Sargassum* showed antibacterial and antioxidant properties [125].

##### Antidepressant Activity

Extracts of *Sargassum swartzii* and *Stoechospermum marginatum* showed significant stimulant and anxiolytic effects on a rat model, which occurred by the amelioration of brain norepinephrine levels [126]. The aqueous extracts of *Cystoseira usneoides, Cystoseira tamarascifolia, Cystoseira nodicaulis, Stypocaulon scoparium,* and *Fucus spiralis* showed antidepressant activity by inhibiting the monoamine oxidase A (MAO-A) enzyme [127].

##### Anticancer Activity

A cytotoxic metabolite, stypoldione, isolated from *Stypodium zonale* inhibited microtubule polymerization, thereby preventing mitotic spindle formation in cell cultures [128]. Ethanolic extracts of *U. pinnatifida* sporophylls induced nonoxidative apoptotic damage on human colon or rectum cancer cells [129]. The derivative of atomaric acid, stypoquinonic acid (Figure 4C), isolated from *S. zonale* is an inhibitor of the tyrosine kinase enzyme. Another atomaric acid derivative, dimethoxy-atomaric acid, showed cytotoxic activity against lung and colon cancer cells [130]. Moreover, fucoidan is known to possess other bioactive properties, such as inhibitory effects on the growth of cancer cells [131]. Sodium alginate (Figure 4J) obtained from brown seaweeds has shown antitumor and anti-inflammatory properties [132]. The anticancer activity was evaluated by analyzing the effects on the cell cycle and apoptosis induction on HepG2 cells [133]. Furthermore, several studies validated the apoptosis induction ability of fucoidans in various tumor cell lines, including melanoma cells, colon cancer, lymphoma, and breast cancer cells [134].

##### Antiangiogenic and Anticoagulant Activities

Phlorofucofuroeckol A (Figure 4I), a phlorotannin (Figure 4H) from *Ecklonia kurome*, exhibited inhibitory activity on the angiotensin-converting enzyme, with an IC_50_ value of 12.74 µM [135]. The sulfated polysaccharides known as fucoidans caused the prevention of cell proliferation and migration and also vascular network formation on human umbilical vein endothelial cells (*HUVEC),* suggesting significant antiangiogenic activity [136]. Remarkably, this effect deteriorated upon the reduction of fucoidan molecular weight (<30 kDa) [137]. An analogous observation was reported for the anticoagulant activities of fucoidans [138]. A fucoidan isolated from *Fucus vesiculosus* showed antithrombotic activity [139].

##### Antiviral Activity

Phlorotannin derivatives extracted from *E. cava*, 8,4′-dieckol and 8,8′-bieckol (Figure 4G), displayed an anti-HIV-1 property by inhibiting the viral reverse transcriptase and the protease at concentrations (IC_50_) of 5.3 and 0.5 μM, respectively [140]. Hayashi et al. studied the fucoidan from *U. pinnatifida* and described its defensive effects against herpes simplex virus (HSV) infections [141]. Also, Queiroz reported on a brown seaweed polysaccharide inhibiting the activity of the reverse transcriptase of HIV [142]. Fucans from *Dictyota mertensii*, *F. vesiculosus*, *Spatoglossum schroederi*, and *Lobophora variegate* show inhibitory effects on the reverse transcriptase of HIV-1 [143].

##### Antiparasitic Activity

Soares demonstrated that brown algae belonging to the family of *Dictyotaceae*, namely *Dictyota pfaffii* and *Canistrocarpus cervicornis,* possess antileishmania activity [144]. Nara reported that the extracts from *Pelvetia babingtonii* and *Fucus evanescens* contain inhibitors of dihydroorotate dehydrogenase, a virulence agent of *Trypanosoma cruzi*, the protozoa responsible of the Chagas disease [145]. Dolabelladienetriol, a diterpene derived from *D. pfaffii,* showed killing effects against *Leishmania* intracellular amastigotes (IC_50_ = 44 µM) as well as anti-HIV-1 activities [146]. Eleganolone (Figure 3), another diterpene from *Bifurcaria bifurcate,* exerted strong inhibitory activity (IC_50_ = 0.53 µg/mL) against the bloodstream forms of *Trypanosoma brucei rhodesiense* [147].

Of course, the clinical application of all these substances depends on further studies and safety evaluations.

#### 3.2.3. Green Seaweeds

Green seaweeds, or *chlorophyta*, are a large group of macroalgae with worldwide distribution. The morphology of some species is presented in Figure 3. Green seaweeds are highly considered for their production of antioxidants, vitamins, and bioactive peptides [148]. Among their bioactive constituents, cell wall polysaccharides, constituting about 38–54% of the seaweed dry matter, show pharmaceutical potential [149]. Some green seaweeds, such as *Caulerpa taxifolia*, *Caulerpa racemose*, and *Cladophora pinnulata*, show hypotensive activities [111]. At the moment, most of the literature on green seaweed products is focused on sulfated polysaccharides because of their interesting properties, including anticoagulant, antioxidant, anticancer, anti-hyperlipidemic, and immunomodulation effects [150]. Ulvan, a sulfated polysaccharide form *Ulva pertusa*, has valuable antioxidant activity [151]. It also acts on the plasma levels of low-density lipoprotein (LDL), high-density lipoprotein (HDL), and triglycerides in mice and can be useful for ischemic, cerebrovascular, and cardiovascular diseases [152]. Sulfated polysaccharides from *U. pertusa*, *Capsosiphon fulvescens,* and *Codium fragile* possess potent immune-modulating activity by stimulating macrophages [153]. Also, the ethanolic extracts of *Codium tomentosum* showed antigenotoxic and antioxidant effects [154] and *Codium decorticatum* showed antibacterial activity [155]. Moreover, methanolic extracts of *Ulva linza,* due to their high polyunsaturated fatty acids (PUFA) content, showed high inhibitory activity against inflammatory response [156].

##### Antiviral Activity

Sulfated polysaccharides from nine different green seaweeds (*Caulerpa brachypus*, *Caulerpa scapelliformis*, *Caulerpa okamurai*, *Chaetomorpha crassa*, *Chaetomorpha spiralis*, *Monostroma nitidum*, *C. fragile*, *Codium adhaerens*, and *Codium latum*) exhibited strong activity against type 1 herpes virus, with the IC_50_ ranging from 0.38 to 8.5 µg/mL, while presenting low cytotoxicity [157]. Rhamnan sulfate, another sulfated polysaccharide from *M. nitidum*, was effective against type 2 herpes virus by inhibiting its adsorption and penetration onto host cells [78]. In a study carried out by Sato et al. (2011), a high-mannose-binding lectin was isolated from *Boodlea coacta*. This lectin showed antiviral activity against HIV-1 infections (EC_50_ *=* 8.2 nM) and influenza viruses [158]. The potent anti-HIV-1 activity was related to the carbohydrate-binding tendency, formerly reported for other antiviral lectins. In addition, ethanolic extracts of *Codium elongatum* and *Ulva fasciata* exhibited antiviral activity against the *Semliki forest* and *Vaccinia* viruses [111].

##### Antioxidant and Anticancer Activities

According to Wang, extracts and monounsaturated fatty acids (MUFA) derivatives from *Ulva lactuca* induced an antioxidant response in cells [159]. Beta-carotene is a potent antioxidant found in green seaweeds and can be accumulated in very high amounts in *Dunaliella salina* [160]. Carotenoids, for example, lutein (Figure 4E) and zeaxanthin from the green seaweed *Chlorococcum humicola*, exhibited antimutagenic activity against benzo[*a*]pyrene-induced mutations in histidine-revertant cells of *Salmonella typhimurium* and were proposed for the reduction of the breast cancer risks [161]. Another carotenoid from green seaweeds, siphonaxanthin (Figure 4D), effectively induced apoptosis in human leukemia (HL-60) cells by caspase-3 activation, accompanied by the modification of growth arrest and DNA-damage-inducible protein (GADD45α), tumor necrosis factor receptors (DR5/TRAIL receptor-2), and Bcl-2 regulatory protein expression pattern [162]. Ganesan reported that siphonaxanthin derived from *C. fragile* possesses considerable antiangiogenic activity [163]. Moreover, the ethanolic extracts of *C. tomentosum* showed antigenotoxic and antioxidant effects [154].

##### Antibacterial and Antifouling Activities

Extracts of *U. fasciata* isolated from the southeast coast of India displayed antibacterial properties and a broad spectrum of antibiotic activity against *B. cereus*, *E. coli*, *B. subtilis*, *Aeromonas hydrophila*, *Vibrio fischeri*, and *Vibrio harveyi* [164]. *Cladophora glomerata* exhibited significant antibacterial activity against the MDR bacterium *Acinetobacter baumannii* and various human and fish pathogens, such as *E. coli*, *B. cereus*, *Vibrio anguillarum*, *V. fischeri, Vibrio parahaemolyticus*, and *Vibrio vulnificus* [165]. An acetylenic sesquiterpene isolated from *Caulerpa prolifera*, caulerpenyne (Figure 4H), exhibited antifouling activity against bacteria and the microalga *Phaeodactylum tricornutum* [166]. Moreover, the ethanolic extracts of *C. decorticatum* showed antibacterial activity [155]

##### Anticoagulant Activity

The earliest report on the anticoagulant effects of substances produced by green seaweeds were carried out on *Codium*, in particular on *C. fragile* ssp. *tomentosoides* [167]. Matsubara also reported on the anticoagulant activity of a sulfated galactan in the *Codium* genus [168]. Furthermore, Maeda described that sulfated polysaccharides from the marine green alga *M. nitidum* yielded a sixfold higher anticoagulant effect than heparin [169]. Also, Synytsya reported on the anticoagulant activity of sulfated polysaccharides derived from *C. fulvescens* [170].

The findings presented above suggest that green marine algae can be considered a promising source of bioactive substances, which should be further studied and exploited for pharmaceutical applications. Some of the biological activities of compounds extracted from marine seaweeds are presented in Table 2.

## 4. Concluding Remarks

The marine environment represents a unique source of bioactive compounds with high pharmaceutical potential. Preclinical and clinical studies are in progress for a number of marine organism derivatives. Nevertheless, several problems should be solved for a deep characterization of biomolecules derived from marine microorganisms, micro, and macroalgae. Notably, marine microbes are notoriously difficult to prepare and maintain in culture. Bacteria likely grow as consortia in the marine environment and dependences on other bacteria for essential nutrients should be guaranteed. These interactions are lacking in isolated laboratory cultures. Further, the marine shear stress, hypersalinity, and antagonists, which induce unique metabolite elaborations, are missing in laboratory cultures. That notwithstanding, these efforts may lead to valuable results. In fact, marine bacteria are significant reservoirs of a plethora of bioactive molecules which have never been found in terrestrial organisms. Moreover, microalgae represent an important, still underestimated source of bioactive metabolites, such as antiviral and anti-AIDS agents. Seaweeds as well produce many different, interesting biologically active substances, such as sulphated polysaccharides, which are promising compounds for drug development [171]. For example, carbohydrate recognition molecules, such as lectins from green seaweeds, have the potential to be used as antitumor and antiviral agents, but they have been rarely investigated [172]. Notably, a natural compound can have variable effects on the human body, as host factors are different. The promising results obtained by an in vitro experiment may not show the same effects in a patient’s body. Moreover, the human body under an inflammatory process presents an activated immune system that can consider even a therapeutic compound a threat. Thus, proper safety assessments of the studied metabolites are required. The optimal dosage determination is very important, as this parameter distinguishes whether a compound will act as a drug or a poison. From a technological point of view, novel cost-effective and large-scale fermentation strategies ought to be devised, and particular attention should be paid to the conditions under which the secondary metabolites are produced. Recreating the physical, chemical, and biological conditions of a marine environment is hardly an achievable task. Metabolic and protein engineering can improve the efficacy of some of the marine candidates with pharmaceutical potential. In the future, the efforts and advances in this direction will certainly open the way for extraordinary discoveries for novel applications of marine-derived compounds in biotechnology and biomedicine.

## Figures and Tables

**Table 1:** Structure and biological activity of some novel marine microorganisms’ natural compounds.

Marine Microorganisms (Bacteria, Fungi, and Cyanobacteria)					
Compound	Chemical Structure	Source/Species	Biological Activity	Mechanism of Action	References
Salinosporamide A	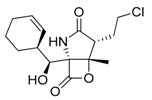	Actinomycete/*Salinispora tropica*	Treatment of multiple myeloma (anticancer); antimalarial	Inhibits proteasome activity by covalently modifying the threonine residue of the active site of the 20S proteasome	[20]
Plinabulin	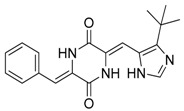	Fungi/*Aspergillus* sp.	Treatment of solid tumors and lymphomas	Depolymerizes microtubules in A549 human lung carcinoma cells	[21]
Alternaramide	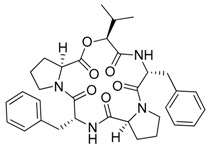	Ascomycete fungi/*Alternaria* sp.	Anti-inflammatory	Inhibits inflammatory mediator expression through TLR4-MyD88-mediated inhibition of NF-кB and MAPK pathway signaling in lipopolysaccharide-stimulated RAW264.7 and BV2 cells	[22]
Macrolactin S	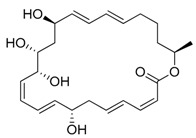	Bacterium/*Bacillus* sp.	Antibacterial	FabG inhibition agent	[23]
Oxaline	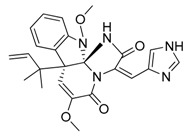	Ascomycete fungi/*Penicillium* sp.	Antitumor	Inhibits cell proliferation and induces cell cycle arrest at the G_2_/M phase in Jurkat cells	[24]
Grassystatin C	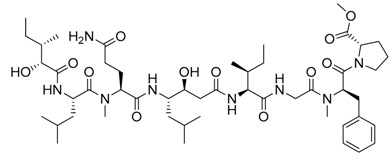	Tropical cyanobacteria/*Okeania lorea*	Cathepsin inhibition	Potent cathepsin E inhibitor that reduces antigen presentation	[25]
Palmyramide A	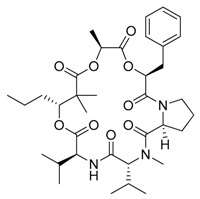	Filamentous cyanobacteria/*Moorea producens*	Antitumor	Sodium channel blocking activity in neuro-2a cells and cytotoxic activity in H-460 human lung carcinoma cells	[26]
Coibamide A	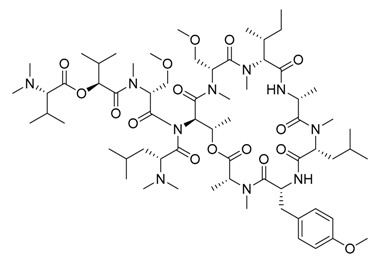	Pantropical cyanobacteria/*Caldora penicillata*	Antitumor cytotoxicity	Inhibits VEGFA/VEGFR2 expression and suppresses tumor growth in glioblastoma xenografts	[27,28]
Hectochlorin	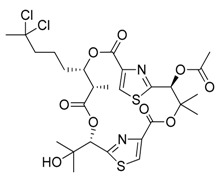	Cyanobacterium/*Lyngbya majusculea* JHB	Cytotoxin, antifungal	Inhibits the growth of human cell lines by hyper-polymerization of actin	[29]
Pompanopeptin A	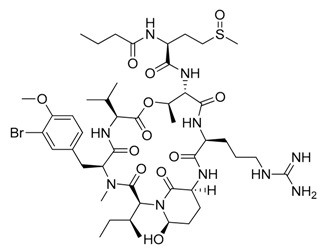	Cyanobacterium/*Lyngbya confervoides*	Trypsin inhibitor	Inhibits trypsin with an IC_50_ value of 2.4 μM; selectivity is conferred by the arginine residue	[30]

**Table 2:** Structure and biological activity of some novel marine seaweeds’ natural compounds.

Natural Compound	Chemical Structure	Species	Biological Activity	Mechanism of action	References
Sulfated galactan	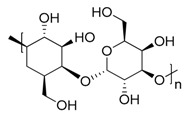	Green alga/*Codium fragile*	Immunostimulating effects via activation of macrophages	Stimulates the production of nitric oxide by inducing iNOS at mRNA and protein levels and induces the expression of several cytokine mRNA, such as IL-1β, IL-6, IL-10, and TNF-α	[71]
Caulerpin	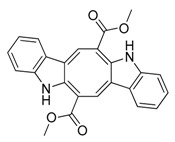	Green alga/*Caulerpa racemosa*	Anti-inflammatory and antinociceptive	Inhibits capsaicin-induced ear edema model and significantly reduces the number of recruited cells	[72]
Pheophytin A	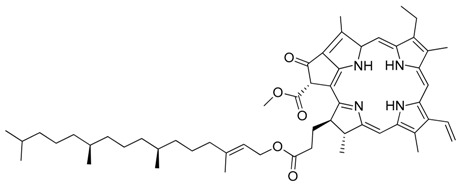	Green alga/*Enteromorpha prolifera*	Anti-inflammatory	Exhibits significant suppression of TPA-induced inflammatory reaction, such as edema formation in BALB/c mouse ear	[73]
Cymopols	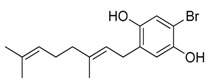	Green alga/*Cymopolia barbata*	Antimutagenic	Inhibits the mutagenicity of 2-aminoanthracene in T-98 strain. Behaves as a metabolic activator	[74]
Caulerpenyne	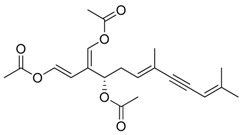	Green alga/*Caulerpa taxifolia*	Anticancer	Shows cytotoxicity in cultured cell lines, such as KB cells and hamster fibroblasts	[75,76]
Fucoxanthin	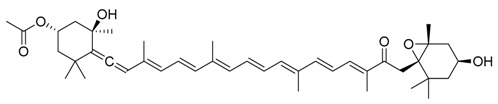	Brown algae	Antidiabetic and antiobesity	Suppresses McP-1, promotes adrb3 and gluT4 expression, and induces uncoupling protein 1 expression in white adipose tissue (WAT) mitochondria, leading to oxidation of fatty acids and heat production in WAT	[77]
Dieckol	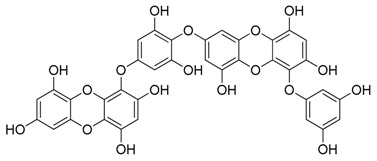	Brown alga/*Ecklonia cava*	Anti-inflammatory and neuroprotective agent	Suppresses LPS-induced production of nitric oxide (NO) and prostaglandin E_2_ (PGE_2_) and the expression of inducible nitric oxide synthase (iNOS) and cyclooxygenase-2 (COX-2) in murine BV2 microglia	[78]
Spiralisone A	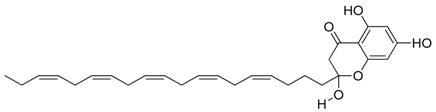	Brown alga/*Zonaria spiralis*	Kinase inhibitor and antibacterial	Shows inhibitory activity against neurodegenerative diseases targeting CDK5/p25, CK1δ, and GSK3β kinases. Inhibits the Gram-positive bacteria *Bacillus subtilis* (ATCC 6051 and 6633)	[79]
Sargaquinoic acid	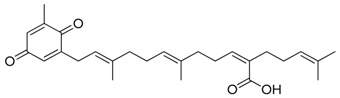	Brown alga/*Sargassum sagamianum*	AChE inhibitor	Inhibits acetylcholinesterase activity	[80]
Phorbasterone B	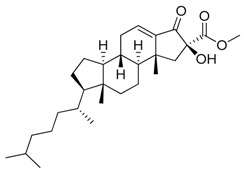	Red seaweed	Antimicrobial	Exhibits antimicrobial activity against *Bacillus cereus, Staphylococcus aureus, Streptococcus pneumoniae*, and *Candida albicans*	[81]
Azocinyl- morpholinone	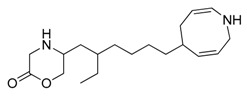	Red seaweed/*Gracilaria opuntia*	Antioxidant, anti-inflammatory by inhibiting cyclooxygenase and lipoxygenase	Azocinyl morpholinone significantly mitigated the carrageenan-induced paw edema	[82]
(5Z)-4-bromo-5-(bromo-methylene)-3-butyl-2(5H)-furanone	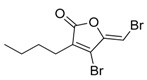	Red seaweed/*Delisea pulchra*	Antifouling agent	Inhibits microbial-induced corrosion related to Gram-positive bacteria	[83]
Kahalalide A	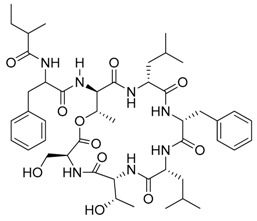	Red seaweed/*Bryopsis* sp.	Antibacterial agent	Shows in vitro activity against *Mycobacterium tuberculosis*	[84,85]
Kahalalide F	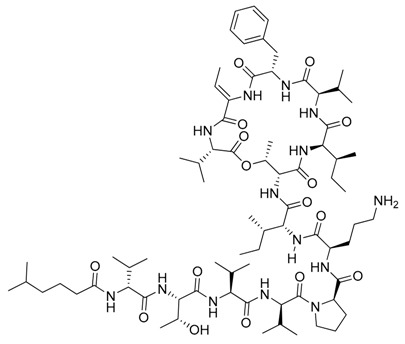	Red seaweed/*Bryopsis* sp.	Antibacterial and anti-HIV agent	Shows antibacterial activity against *M. tuberculosis* and proposed for the treatment of lung cancer	[84,85]
